# Supplementary material for: Recurrence Kinetics after Laparoscopic Versus Open Surgery in Colon Cancer. A Meta-Analysis
Source: J Clin Med. 2021 Sep 15;10(18):4163. doi: 10.3390/jcm10184163 (PMC8466495; doi:10.3390/jcm10184163)
Supplement: Supplementary file 1 [file jcm-10-04163-s001.zip › jcm-1371660-supplementary.pdf]

Supplementary S1. Databases and Search Strategies.

Ovid Medline (1947-Present)

#1 – MeSH descriptor: [Colorectal Neoplasms] explode all trees

#2 – Colon.tw

#3 – Colorect.tw

#4 – sigmoid.tw

#5 – bowel.tw

#6 – large intestine.tw

#7 – cecum.tw

#8 – caecum.tw

#9 – cancer.tw

#10 – neoplas.tw

#11 – tumo.tw

#12 – carcino.tw

#13 – adenocarcino.tw

#14 – MeSH descriptor: [Laparoscopy] explode all trees

#15 – laparoscop.tw

#16 – MeSH descriptor: [Laparotomy] explode all trees

#17 – laparotomy.tw

#18 – open surgery.tw

#19 – randomi.tw

#20 - #2 **OR** #3 **OR** #4 **OR** #5 **OR** #6 **OR** #7 **OR** #8

#21 - #9 **OR** #10 **OR** #11 **OR** #12 **OR** #13

#22 - #20 **AND** #21

#23 - #14 **OR** 15

#24 - #16 **OR** #17 **OR** #18

#25 - #1 **OR** # 22

#26 - #19 **AND** #23 **AND** #24 **AND** #25

Additional Filters:

Randomized Controlled Trial; Humans

Cochrane Library

#1 – MeSH descriptor: [Colorectal Neoplasms] explode all trees

#2 – Colon [tw]

#3 – Colorect[tw]

#4 – sigmoid[tw]

#5 – bowel.tw

#6 – large intestine [tw]

#7 – cecum [tw]

#8 – caecum [tw]

#9 – cancer [tw]

#10 – neoplas [tw]

#11 – tumo [tw]

#12 – carcino [tw]

#13 – adenocarcino [tw]

#14 – MeSH descriptor: [Laparoscopy] explode all trees

#15 – laparoscop [tw]

#16 – MeSH descriptor: [Laparotomy] explode all trees

#17 – laparotomy [tw]

#18 – open surgery [tw]

#19 – randomi [tw]

#20 - #2 **OR** #3 **OR** #4 **OR** #5 **OR** #6 **OR** #7 **OR** #8

#21 - #9 **OR** #10 **OR** #11 **OR** #12 **OR** #13

#22 - #20 **AND** #21

#23 - #14 **OR** 15

#24 - #16 **OR** #17 **OR** #18

#25 - #1 **OR** # 22

#26 - #19 **AND** #23 **AND** #24 **AND** #25

Additional Filters:

Trials; Humans

PubMed Central

[(Colorectal Neoplasms) OR ((colon.tw **OR** colorect.tw **OR** sigmoid.tw **OR** bowel.tw **OR** "large intestine".tw **OR** cecum.tw OR caecum.tw**) AND (**cancer.tw OR neoplas.tw **OR** tumo.tw OR carcino.tw **OR** adenocarcin.tw**))]**

AND *(laparoscopy* ***OR*** *laparoscop.tw)*

AND *(Laparotomy* ***OR*** *laparotom.tw* ***OR*** *open surgery.tw)*

Additional Filters:

Randomized Controlled Trial

EMBASE (1974 – 2020 week 20)

#**1** – exp Colorectal Neoplasms/

#**2** – Colon.tw

#**3** – Colorect.tw

#**4** – sigmoid.tw

#**5** – bowel.tw

#**6** – large intestine.tw

#**7** – cecum.tw

#**8** – caecum.tw

#**9** – cancer.tw

#**10** – neoplas.tw

#**11** – tumo.tw

#**12** – carcino.tw

#**13** – adenocarcino.tw

#**14** – exp Laparoscopy/

#**15** – laparoscop.tw

#**16** – exp Laparotomy/

#**17** – laparotomy.tw

#18 – open surgery.tw

#19 – randomi.tw

#20 - #2 **OR** #3 **OR** #4 **OR** #5 **OR** #6 **OR** #7 **OR** #8

#21 - #9 **OR** #10 **OR** #11 **OR** #12 **OR** #13

#22 - #20 **AND** #21

#23 - #14 **OR** 15

#24 - #16 **OR** #17 **OR** #18

#25 - #1 **OR** # 22

#26 - #19 **AND** #23 **AND** #24 **AND** #25

Additional Filters:

Randomized Controlled Trial; Humans

Supplementary S2. Independently Developed Data Extraction Tool.

Data extraction template				
Review:				
Review author:				
**ADMINISTRATION DETAILS**				
Study ID				
Date of data extraction				
Publication status				
**AIM OF THE STUDY**				
**STUDY ELIGIBILITY**				
**STUDY DETAILS**				
Author				
Year				
Country				
Language				
**METHODS**				
Design				
Setting of the study				
Duration of the study				
Follow up period				
Co-intervention				
**ELIGIBILITY CRITERIA FOR THE STUDY**				
Inclusion criteria				
Exclusion criteria				
**PARTICIPANTS (include all p-**				
**Patient baseline characteristics**	**Total**	**Intervention**	**Comparator/**	**Difference**
Number randomised				
Number analysed				
Follow up time				
Number of withdrawal, lost to follow				
Age (years): (specify mean/median,				
Gender (M/F), n (%)				
INTERVENTIONS/COMPARATORS				
**Intervention**				
Type of laparoscopic technique eg. hand assisted				
Number converted from laparoscopic to open				
**Comparator**				
EFFICACY OUTCOMES (Include all p-values)				
n/N (%), mean (SD), median (range)	**Intervention**		**Comparator /Control group**	
score	**Events OR**	**Total no.**	**Events OR**	**Total no. analysed**
3 year DFS				
3 year OS				
5 year DFS				
5 year OS				
**STATISTICAL ANALYSIS** e.g. ITT				
ADVERSE EVENTS	**Intervention**		**Comparator**	
**POTENTIAL CONFOUNDERS**				
**Additional information and/or Comments**				

Table S1. Summary of critical appraisals of selected studies, performed using the SIGN checklist [25].

	Braga et al., 2010	Braga et al., 2005	Chung et al., 2007	COLOR trial, 2009	COST trial, 2007	Ishibe et al., 2017	JCOG0404, 2017	Li et al., 2012	Toritani et al., 2019	Tung et al., 2013
The study addresses an appropriate and clearly focused question.	Y	Y	Y	Y	Y	Y	Y	Y	Y	Y
The assignment of subjects to treatment groups is randomised.	Y	Y	Y	Y	Y	Y	Y	Y	Y	Y
An adequate concealment method is used.	Y	Y	Y	Y	CS	CS	CS	Y	Y	Y
The design keeps subjects and investigators 'blind' about treatment allocation.	N	N	CS	N	N	N	N	N	N	CS
The treatment and control groups are similar at the start of the trial.	Y	Y	Y	Y	Y	Y	Y	Y	Y	Y
The only difference between groups is the treatment under investigation.	N	N	CS	N	N	N	N	Y	N	Y
All relevant outcomes are measured in a standard, valid and reliable way.	Y	Y	Y	Y	Y	Y	Y	Y	Y	Y

What percentage of the individuals or clusters recruited into each treatment arm of the study dropped out before the study was completed?	LS: 0%<br><br>OS: 0%	LS:2.24%<br><br>OS:2.24%	LS:0%<br><br>OS:0%	LS:2%<br><br>OS:2%	LS:1.15%<br><br>OS:0.92%	LS:2%<br><br>OS:8%	LS:5.3%<br><br>OS:6.8%	LS:1.41 %<br><br>OS:1.35 %	LS: 0%<br><br>OS: 0%	LS: 8.3%<br><br>OS:45.8 %
All the subjects are analysed in the groups to which they were randomly allocated (often referred to as intention to treat analysis).	Y	Y	Y	Y	Y	Y	Y	Y	Y	CS
Where the study is carried out at more than one site, results are comparable for all sites.	NA	NA	NA	Y	CS	NA	CS	NA	NA	NA
How well was the study done to minimise bias?	++	++	++	++	+	+	++	++	++	+
Taking into account clinical considerations, your evaluation of the methodology used, and the statistical power of the study, are you certain that the overall effect is due to the study intervention?	Y	Y	Y	Y	Y	Y	Y	Y	Y	Y

Are the results of this study directly applicable to the patient group targeted by this guideline?	Y	Y	Y	Y	Y	Y	Y	Y	Y	Y

KEY	
Y	Yes
N	No
CS	Cannot say from paper
NA	Not applicable to paper

Table S2. Summary of Revised Cochrane Risk-of Bias Tool for Randomised Trials (RoB 2.0) [26].

	Braga et al., 2005	Braga et al., 2010	Chung et al., 2007	COLOR, 2009	COST, 2007	Ishibe et al., 2017	JCOG404., 2017	Li et al., 2012	Toritani et al., 2019	Tung et al., 2013
Risk of bias arising from the randomisation process	LR	LR	LR	LR	LR	LR	SC	LR	LR	HR
Risk of bias due to deviations from the intended interventions (effect of assignment to intervention)	HR	HR	HR	HR	SC	HR	HR	SC	HR	SC
Risk of bias due to deviations from the intended interventions (effect of adhering to intervention)	HR	HR	SC	HR	SC	HR	LR	LR	HR	SC
Risk of bias due to missing outcome data	LR	LR	LR	LR	LR	LR	LR	LR	LR	HR
Risk of bias in measurement of the outcome	LR	LR	LR	LR	LR	SC	LR	LR	SC	LR

KEY	
LR	Low Risk
HR	High Risk
SC	Some Concerns

Risk of bias in the selection of the reported result	LR	LR	LR	LR	LR	LR	LR	LR	LR	LR
Overall risk of bias	HR	HR	HR	HR	SC	HR	HR	SC	HR	HR
